# Endothelial Activation Microparticles and Inflammation Status Improve with Exercise Training in African Americans

**DOI:** 10.1155/2013/538017

**Published:** 2013-04-18

**Authors:** Dianne M. Babbitt, Keith M. Diaz, Deborah L. Feairheller, Kathleen M. Sturgeon, Amanda M. Perkins, Praveen Veerabhadrappa, Sheara T. Williamson, Jan Kretzschmar, Chenyi Ling, Hojun Lee, Heather Grimm, Sunny R. Thakkar, Deborah L. Crabbe, Mohammed A. Kashem, Michael D. Brown

**Affiliations:** ^1^Hypertension, Molecular and Applied Physiology Laboratory, Department of Kinesiology, Temple University, 1800 N. Broad Street, Philadelphia, PA 19122, USA; ^2^Center for Behavioral Cardiovascular Health, Department of Medicine, Columbia University Medical Center, New York, NY 10027, USA; ^3^ECRI Institute, Health Technology Assessment Group, Plymouth Meeting, PA 19462, USA; ^4^Institute of Translational Medicine and Therapeutics, University of Pennsylvania, Philadelphia, PA 19104, USA; ^5^Department of Kinesiology, Missouri State University, Springfield, MO 65897, USA; ^6^Department of Exercise Science, College of Education, Shippensburg University, Shippensburg, PA 17257, USA; ^7^Vascular Health Laboratory, Department of Kinesiology & Nutrition, University of Illinois at Chicago, Chicago, IL 60607, USA; ^8^Division of Cardiology, Department of Medicine, School of Medicine, Temple University, Philadelphia, PA 19140, USA

## Abstract

African Americans have the highest prevalence of hypertension in the world which may emanate from their predisposition to heightened endothelial inflammation. The purpose of this study was to determine the effects of a 6-month aerobic exercise training (AEXT) intervention on the inflammatory biomarkers interleukin-10 (IL-10), interleukin-6 (IL-6), and endothelial microparticle (EMP) CD62E+ and endothelial function assessed by flow-mediated dilation (FMD) in African Americans. A secondary purpose was to evaluate whether changes in IL-10, IL-6, or CD62E+ EMPs predicted the change in FMD following the 6-month AEXT intervention. A pre-post design was employed with baseline evaluation including office blood pressure, FMD, fasting blood sampling, and graded exercise testing. Participants engaged in 6 months of AEXT. Following the AEXT intervention, all baseline tests were repeated. FMD significantly increased, CD62E+ EMPs and IL-6 significantly decreased, and IL-10 increased but not significantly following AEXT. Changes in inflammatory biomarkers did not significantly predict the change in FMD. The change in VO_2 max_ significantly predicted the change in IL-10. Based on these results, AEXT may be a viable, nonpharmacological method to improve inflammation status and endothelial function and thereby contribute to risk reduction for cardiovascular disease in African Americans.

## 1. Introduction

The most recent report (May 2012) from the World Health Organization, as well as the preponderance of published articles on hypertension and race, supports the conclusion that African Americans have the highest prevalence of hypertension in the world. Research has demonstrated that African Americans have a greater prevalence of endothelial dysfunction when compared to their Caucasian counterparts, and researchers report that they suspect that this predisposes them to hypertension [1, 2]. Hypertension is a result of independent and interactive effects from multiple genetic and environmental factors. Inflammation of the endothelium, a pathological mechanism that can cause endothelial dysfunction and a precursor to hypertension, has been identified as one of these factors.

It is thought that the balance between pro- and anti-inflammation plays a crucial role as a determinant of endothelial homeostasis and health [3]. Bautista reviewed multiple studies and reported a positive association between hypertension and some proinflammatory markers including C-reactive protein (CRP), interleukin-6 (IL-6), and tumor necrosis factor alpha (TNF-*α*) [4]. Experimental evidence has also established that several proinflammatory cytokines, including IL-6, contribute to endothelial dysfunction which may lead to increased peripheral vascular resistance and consequently hypertension [5]. In contrast, interleukin-10 (IL-10) is a multifunctional cytokine that inhibits activation and the effector function of  T cells, monocytes, and macrophages and ultimately terminates inflammatory responses [6]. Elevated circulating levels of IL-10 are associated with improved endothelial function in individuals with ongoing systemic inflammation [7] and in coronary artery disease (CAD) patients [3].

Brachial artery flow-mediated dilation (FMD) is the conventional method used to assess endothelial function and health in humans because of its high feasibility as a noninvasive, ultrasound testing modality. Its evaluation is thought to be an important index in subjects at risk for cardiovascular disease (CVD) that may contribute to understanding the extent of the inflammatory status of the endothelium [8]. In recent years, evidence suggests that endothelial activation, characterized by increased inflammation, is an early event in endothelial dysfunction and may be identified with the endothelial microparticle (EMP) inducible marker CD62E+ which is sensitive to endothelial activation [9]. Experimental evidence as reported in a review article by Boulanger et al. suggests that plasma levels of EMPs may be a specific marker of endothelial dysfunction in patients with CVD and may provide further information regarding the status of the endothelium beyond vasodilation [10]. Therefore, the detection and quantification of EMPs may be a valuable tool to assess cardiovascular risk.

Increased blood flow shear stress during aerobic exercise has been associated with favorable endothelial adaptations [11]. Additionally, chronic aerobic exercise has been demonstrated to improve the plasma inflammatory status, including IL-6 and IL-10, in certain populations. Aerobic exercise training (AEXT) may lead to the adaptive response of increasing plasma IL-10 concentrations and decreasing both plasma IL-6 concentrations and CD62E+ EMPs in African Americans, thereby improving endothelial function by reducing inflammation. However, despite the high prevalence of hypertension and CVD in African Americans, few studies have investigated the effects of AEXT on inflammation and endothelial health in an effort to develop preventive measures to reduce the CVD disease burden among this high-risk population. Therefore, the purpose of this study was to determine the effects of a 6-month AEXT intervention on plasma levels of IL-10, IL-6, and CD62E+ EMPs and endothelial function assessed by FMD in a cohort of middle-to-older-aged African Americans. Furthermore, a secondary purpose was to evaluate whether changes in IL-10, IL-6, or CD62E+ EMPs predicted the change in FMD following the 6-month AEXT intervention. 

## 2. Methods

This study employed a pre-post design following the completion of screening and dietary stabilization. Sedentary, putatively healthy, middle-to-older-aged (40–75 y/o) African American men and women were recruited and underwent a series of screening tests to ensure that they were free of disease and conditions that may confound interpretation of results. All qualified participants then completed a dietary stabilization period in order to control for the effects of interindividual variations in dietary intake. Finally, any participants using antihypertensive monotherapy were appropriately tapered from their medication, and suspension of medication was continued for the duration of the study. This was done to avoid an AEXT by medication interactive effect. Following dietary stabilization and a minimum of 2 weeks after medication tapering, baseline testing was conducted. This included office blood pressure measurements, FMD studies, fasting blood sampling, and graded exercise testing. FMD studies and fasting blood sampling were conducted on separate days but under the same conditions. Upon completion of baseline testing, participants engaged in a 6-month AEXT intervention under the direct supervision of laboratory personnel. At the conclusion of the 6-month intervention, participants repeated all baseline tests. 

### 2.1. Participants

Participants were required to be between the ages of 40–75 years inclusively, sedentary (self-reported, regular aerobic exercisers ≤ 2 days per week), nondiabetic (fasting blood glucose ≤ 126 mg/dL), nonsmoking (≥2 years), have a clinic blood pressure <160/100 mmHg (i.e., not stage II hypertensive), and have no documented history of CVD, hypercholesterolemia (total cholesterol > 240 mg/dL), renal disease, or pulmonary disease. Participants on lipid lowering medications, medications that affect cardiovascular or renal hemodynamics, or who were taking more than one antihypertensive medication were excluded from this study. Both premenopausal and postmenopausal (self-reported absence of menses) women were included in the study. All postmenopausal women were required to continue their hormone replacement therapy, either on or off, for the duration of the study. These inclusion criteria were used to create a more homogeneous group of middle-to-older-aged African Americans who were at low-to-moderate risk for CVD but who were otherwise putatively healthy. Each participant gave written informed consent following a complete explanation of the study during their first laboratory visit. The protocol was approved by the Temple University Institutional Review Board.

### 2.2. Screening

Eligibility of all qualified participants was ensured via completion of three screening visits prior to inclusion in the study. Screening visit one followed a 12-hour postabsorptive single blood sampling to assess blood chemistries and a urinalysis to assess renal function. Any individual with a total cholesterol >240 mg/dL or fasting blood glucose >126 mg/dL was excluded from the study. Estimated glomerular filtration rate (eGFR) was calculated using the four-variable modification of diet in renal disease (MDRD) study equation specific to African Americans. Any participant who exhibited evidence of renal disease (eGFR < 60 mL min^−1^ per 1.73 m^2^) was excluded from the study.

Screening visits two and three required all qualified participants to undergo a physician-administered physical examination and a cycle ergometer echocardiogram stress test to confirm that participants displayed no evidence of latent cardiovascular, pulmonary, or other chronic diseases.

### 2.3. Plasma IL-10 and Plasma IL-6 Concentration

 Blood samples were collected in the morning following a 12-hour overnight fast. Blood was drawn into EDTA tubes, centrifuged at 2,000 g for 20 minutes at 4°C, and then the plasma was frozen at −80°C until the time of the assay. Concentrations of IL-10 and IL-6 were determined using an enzyme-linked immunosorbent assay (R & D Systems, Minneapolis, MN, USA). Assays were conducted and analyzed according to manufacturer's protocol. Absorbance was recorded using a Spectra Max Microplate Reader (Molecular Devices, Sunnyvale, CA, USA). The plate was read at 490 nm with correction for optical imperfections at 650 nm for IL-10 and at 450 nm with correction for optical imperfections at 540 nm for IL-6. Intraassay and interassay CVs were 5.5% and 11.9%, respectively, for IL-10 and 7.4% and 4.5%, respectively, for IL-6.

### 2.4. CD62E+ Endothelial Microparticles Identification and Quantification

Circulating EMPs were quantified using a venous blood sample obtained from the antecubital vein in the morning following a 12-hour overnight fast. Samples were collected into EDTA tubes using a 21-gauge needle and were centrifuged at 2,000 g for 20 minutes at 4°C immediately after collection to separate plasma from whole blood. Plasma samples were then stored at −80°C until measurement. On the day of analysis, two sequential centrifugation steps were used to reduce background signals contributed by plasma proteins and residual contaminating/unwanted cells and to concentrate microparticles in order to improve the signal-to-noise ratio during flow cytometric analysis. First, plasma samples were thawed and centrifuged at 1,500 g for 20 minutes at room temperature to obtain platelet poor plasma (PPP). The top two-thirds volume of PPP were then transferred to a new tube and further centrifuged at 1,500 g for 20 minutes at room temperature to obtain cell-free plasma. The supernatant was used for microparticle analysis. A volume of 100 *μ*L supernatant was incubated with fluorochrome-labeled antibodies for 20 minutes at room temperature in the dark and then was fixed by adding 93 *μ*L of 10% formaldehyde. The mixture was protected from light and incubated while being gently mixed using a shaker for 20 minutes. The antibody CD62E-PE (15 *μ*L per sample) was used to distinguish EMP subpopulations. All antibodies were obtained from BD Biosciences. After antibody incubation, samples were diluted with 500 mL of 0.22 *μ*m double-filtered PBS before flow cytometric analysis. Two additional samples were also prepared to serve as negative controls and as a calibration. For the negative control tube, 733 *μ*L of PBS was added to one tube. The calibrator sample was prepared using two drops of 0.9 *μ*m standard precision NIST traceable polystyrene particle beads (Polysciences Inc, Warrington, PA, USA), and was added to PBS according to the manufacturer's instructions. All samples were immediately analyzed by flow cytometry.

Samples and controls were analyzed using a BDLSRII flow cytometer (BD Biosciences, San Jose, CA, USA) and BD FACSDIVA software (v 1.2.6; BD Biosciences). Forward scatter scale, side scatter scale, and each fluorescent channel were set in logarithmic scale. Events included in the set gate (<1.0 *μ*m) were identified in forward and side scatter intensity dot representation and plotted on 2-color fluorescence histograms. CD62E+ events <1.0 *μ*m were defined as EMPs. Fluorescence minus one control and nonstained samples were used to discriminate true events from noise and to increase the sensitivity for microparticle detection for each sample. The flow rate was set on medium, and all samples were run for 180 seconds. Using beads, medium flow rate was calculated, and a mean sample volume of 101 *μ*L per 180 seconds was processed. EMPs were expressed as events per *μ*L plasma.

### 2.5. Brachial Artery Flow-Mediated Dilation

FMD was measured as a percent difference between the diameter of the brachial artery during basal conditions and the diameter of the artery following reactive hyperemia. Brachial artery diameter was measured in response to increased flow. All measurements were performed in the morning following a 12-hour overnight fast during which time participants refrained from food, drink (with the exception of water), caffeine, alcohol, antihistamines, and anti-inflammatory medications. A 7.5 MHz linear phased array ultrasound transducer attached to a Sonos 5500 ultrasound machine (Philips Medical Systems, Bothell, WA, USA) was used to image the brachial artery longitudinally. An electrocardiogram (ECG) was continuously monitored. All measurements of brachial artery diameter and blood velocity were measured by a trained cardiologist after the participant rested in a quiet and dim room at a controlled ambient temperature of 20–26°C for a minimum duration of 10 minutes. The participant's right arm was comfortably immobilized in the extended position to allow for ultrasound scanning of the brachial artery 5–10 cm above the antecubital fossa. Simultaneous doppler measurements for blood velocity and 2D ultrasound imaging for right brachial artery diameter were continuously recorded for 2 minutes at baseline. After recording of baseline images, reactive hyperemia was induced by distal occlusion of the vessel using a cuff inflated to a suprasystolic pressure (200 mmHg) for 5 minutes on the right forearm and distal to the antecubital fossa. Brachial artery diameter was then recorded at 1-minute postcuff release at a fixed distance from an anatomic marker at the end of diastole. 

### 2.6. Aerobic Exercise Training Intervention

A submaximal graded exercise test was performed to determine participants' cardiovascular fitness and to develop individualized exercise prescriptions for the AEXT intervention. A modified Bruce protocol submaximal treadmill exercise test was performed with continuous measurement of breath-by-breath gas sampling oxygen consumption (VO_2_) using a calibrated metabolic cart (Vmax Encore, SensorMedics, Yorba Linda, CA, USA). ECG was continuously monitored, and the treadmill test was terminated when the participant reached 75–80% of their predicted heart rate reserve. A standard regression formula using data collected by indirect calorimetry (VO_2_ averaged over each 60-second period) and ECG (minute heart rates) was used to predict VO_2max⁡_, a measure of cardiovascular fitness, as recommended by the American College of Sports Medicine Guidelines for Exercise Testing and Prescription.

Participants engaged in a 24-week AEXT intervention under direct supervision of lab personnel 3x/week, beginning with 20 minutes of exercise/session at 50% of VO_2max⁡_. Training duration was then increased by 5 minutes each week until 40 minutes of exercise at 50% of VO_2max⁡_ was reached. Training intensity was then increased by 5% each week until 65% of VO_2max⁡_ was achieved. At week 8, participants reached the desired exercise duration and intensity of 40 minutes at 65% of VO_2max⁡_, which they maintained as their prescription for the remainder of the study. Exercise modes included treadmill walking/jogging, stair stepping, stationary cycling, rowing ergometry, arm ergometry, and elliptical cross-training. To monitor exercise intensity, participants were instructed on how to use heart rate monitors. Study personnel recorded participants' exercise mode, heart rate, and duration in printed logs to ensure adherence to the prescribed exercise training program. Heart rate was recorded every 10 minutes. At week 12, participants completed a second submaximal treadmill exercise test as a basis for adjustment of their exercise prescription to account for changes in cardiovascular fitness. The gradual progression of training duration and intensity was used in order to avoid excessive fatigue and musculoskeletal complaints, thereby maximizing adherence. 

### 2.7. Statistical Analyses

Among the 42 participants who completed the 6-month AEXT intervention, the data used in the statistical analysis for each primary outcome variable were FMD testing (*n* = 26), CD62E+ EMPs (*n* = 28), IL-6 (*n* = 32), and IL-10 (*n* = 26). The differences in each variable sample size are related to issues with participant scheduling, acquiring blood samples, or assay procedure.

Data are expressed as mean ± the standard error of the mean (SEM). The distribution of all variables was examined using the Shapiro-Wilk test of normality. Pre-AEXT and post-AEXT were compared using the paired samples Wilcoxon signed-rank test. Simple linear regression was used to calculate relationships between the variables. Statistical significance was set at a *P* value of <0.05. All statistical analyses were performed using SPSS version 19.0 (SPSS Inc., Chicago, IL, USA).

## 3. Results

### 3.1. Laboratory Values of Participants before and after Exercise Training

The study group consisted of 42 African American men (*n* = 6; 14.3%) and women (*n* = 36; 85.7%). The mean age of the group was 52.7 ± 1.0 years. The laboratory values of the participants measured prior and subsequent to the AEXT intervention are presented in Table 1. The 6-month AEXT intervention significantly increased VO_2max⁡_ and significantly decreased BMI, plasma triglycerides, and fasting blood glucose. Total cholesterol, LDL cholesterol, HDL cholesterol, and mean systolic and diastolic blood pressure were not significantly changed following the AEXT intervention. 

### 3.2. Endothelial Function before and after Exercise Training

**Figure 1:**
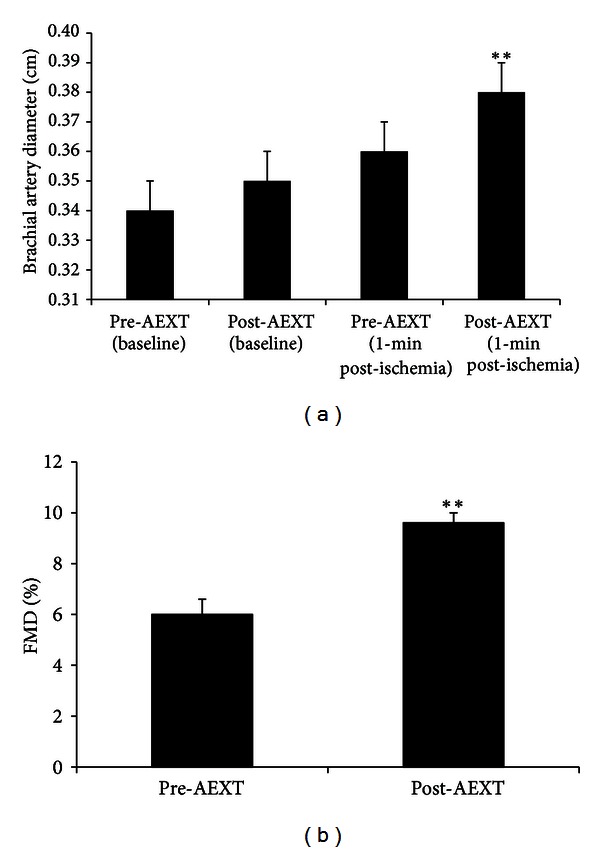
Measures of brachial artery diameter and endothelial function before and after AEXT. The upper panel (a) shows brachial artery diameter at baseline and at 1-minute post-ischemia pre- and post-AEXT. The lower panel (b) shows FMD% pre- and post-AEXT. Bars are expressed as mean ± SEM. **Denotes significant differences pre- versus post-AEXT; *P* < 0.01.

Pre- and post-AEXT values of measures obtained from assessment of endothelial function by FMD testing are presented in Figure 1. There was a 2.9% increase in baseline brachial artery diameter (Figure 1(a)) following AEXT; however, this increase was not statistically significant. Brachial artery diameter 1-minute post-ischemia was significantly increased by 5.6% (Figure 1(a)) following AEXT. The relative increase in brachial artery diameter from baseline to post-ischemia (FMD%) was significantly increased by 60% (Figure 1(b)) following AEXT. 

### 3.3. Inflammatory Biomarkers before and after Exercise Training

**Figure 2:**
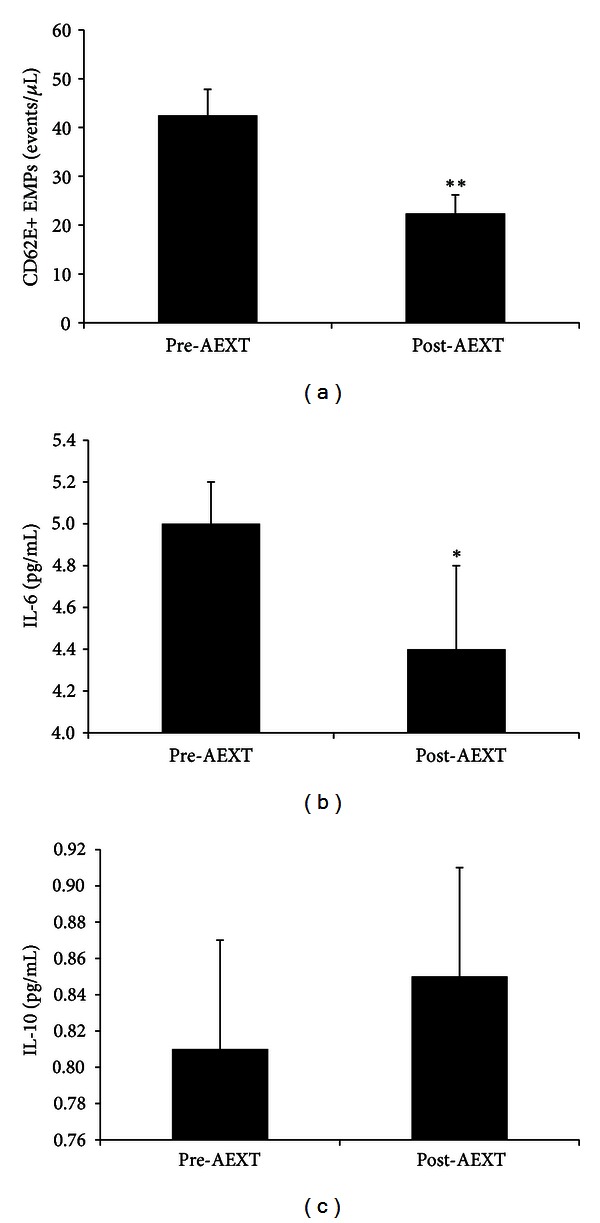
Inflammatory biomarkers before and after AEXT. The upper panel (a) shows CD62E+ EMPs pre- and post-AEXT. The middle panel (b) shows IL-6 pre- and post-AEXT. The lower panel (c) shows IL-10 pre- and post-AEXT. Bars are expressed as mean ± SEM. *Denotes significant differences pre- versus post-AEXT; *P* < 0.05. **Denotes significant differences pre- versus post-AEXT; *P* < 0.01.

Pre- and post-AEXT values for the inflammatory biomarkers are presented in Figure 2. The 6-month AEXT intervention elicited statistically significant changes in CD62E+ EMPs and IL-6. There was a significant 47.3% decrease in CD62E+ EMPs (Figure 2(a)) and a significant 12% decrease in IL-6 (Figure 2(b)) following AEXT. IL-10 was increased by 4.9% (Figure 2(c)) following AEXT; however, it was not statistically significant. 

### 3.4. Regression Analyses

Simple linear regression using change values for each biomarker revealed that changes in CD62E+ EMPs, IL-10, or IL-6 did not significantly predict the change in FMD%. Based on the combined *r* ^2^ values, CD62E+ EMPs, IL-10, and IL-6 accounted for 10.3% of the change in FMD%. Simple linear regression demonstrated that the change in VO_2max⁡_ significantly predicted the change in IL-10 (*n* = 25; *P* = 0.02).

## 4. Discussion

 The primary findings of the present study demonstrated that 6 months of AEXT elicited significant positive improvements in the inflammatory biomarkers IL-6 and CD62E+ EMPs, as well as the endothelial function marker FMD in a cohort of middle-to-older-aged African Americans. Other studies that measured inflammatory biomarkers and endothelial function prior to and subsequent to AEXT have demonstrated similar results, but to our knowledge this is the first study that measured all of these complementary biomarkers prior to and subsequent to AEXT in an African American population. 

 Improvements in FMD following AEXT have been well documented in previous research. Cornelissen et al. demonstrated a significant increase in FMD% following 12 weeks of aerobic exercise in stable CAD patients [12]. Similarly, Luk et al. conducted research to determine the effect of 8 weeks of AEXT on FMD in patients with stable CAD and demonstrated significant improvements in FMD [13]. Furthermore, Nualnim et al. reported that 12 weeks of regular swimming exercise in a group of putatively healthy adults with prehypertension or stage 1 hypertension significantly improved FMD [14]. However, none of these published studies included sufficient numbers of African Americans to draw any conclusions about the effect of AEXT on FMD in this population. The present study provides some evidence that AEXT may also be beneficial for improving FMD in African Americans.

The results of the present study demonstrated that the change in inflammatory biomarkers CD62E+ EMPs, IL-6, and IL-10 together accounts for 10.3% of the change in FMD% following AEXT. These findings suggest that the three inflammatory biomarkers measured may be contributory to the health of the endothelium; however, there are other factors that may also impact overall endothelial health. It is possible that other biomarkers that were not the focus of this study such as C-reactive protein, oxidized LDL, vascular adhesion molecule, or von Willebrand factor may be better predictors of the change in FMD% with AEXT in this population.

To our knowledge, the effect of AEXT on CD62E+ EMPs has not been previously investigated in any population. CD62E+ EMPs have been identified as markers of inflammatory endothelial cell activation [9, 10, 15, 16]. Therefore, the detection and quantification of EMPs may be a valuable marker in the early detection of cardiovascular risk. Lee et al. demonstrated that a high level of CD62E+ EMPs is associated with cardiovascular events in patients with a history of stroke, suggesting that systemic endothelial activation is associated with the risk for cardiovascular morbidities [17]. The present study provides some of the first evidence that AEXT may attenuate endothelial activation in African Americans which may have clinical importance given the recent findings from Lee et al.

IL-6 is a pleiotropic cytokine whose primary biological functions include mediation of proinflammatory responses and cytoprotection [18]. It is released by endothelial cells in response to inflammatory stress and is essential in the pathogenesis of vascular inflammatory diseases [19, 20]. A review of multiple studies by Boos and Lip concluded that IL-6 contributes to endothelial dysfunction which may lead to increased peripheral vascular resistance and consequently hypertension [5]. The effect of AEXT on circulating levels of IL-6 following AEXT has been previously investigated. Beckie et al. demonstrated that there were significant reductions in IL-6 concentrations in women with CAD following a cardiac rehabilitation exercise program [21]. Additionally, a meta-analysis by Swardfager evaluated changes in inflammatory biomarkers subsequent to exercise interventions in patients with CAD and demonstrated significant decreases in plasma IL-6 concentration [22]. The present study provides further evidence that AEXT may attenuate plasma IL-6 concentrations, and to our knowledge, demonstrating such for the first time in an African American population.

IL-10 is an anti-inflammatory cytokine produced by immune and nonimmune cells [23]. Its primary biological function is to attenuate inflammatory responses, and it has an anti-inflammatory effect on monocyte/endothelium interactions [6, 24]. IL-10 has been demonstrated to participate in preserving endothelial function during acute inflammation [25]. Although a clear mechanism has not yet been elucidated, emerging evidence suggests that IL-10 has a role in vascular protection [23]. Most studies that have measured serum or plasma IL-10 concentrations have found detectable levels of IL-10 in a diseased population versus healthy subjects. Blay et al. measured IL-10 in 153 subjects with non-Hodgkin's lymphoma and compared them to a control group of 60 healthy subjects. The researchers found that IL-10 was not detectable in any of the healthy subjects, but it was detectable in about half of the diseased subjects [26]. In the present study, the fact that we were able to detect IL-10 even at relatively low levels in our putatively healthy African American population may be interpreted as a result of the increased predisposition in this population to chronic low-grade inflammation.

Several studies have previously examined the effect of AEXT on circulating levels of IL-10. Ribeiro et al. examined the effect of AEXT on the plasma inflammatory status of post-myocardial infarction patients and concluded that AEXT increased IL-10, suggesting enhancement of anti-inflammation [27]. Furthermore, Goldhammer et al. demonstrated that AEXT in CAD patients was effective in increasing IL-10, leading to improvements in coronary risk status [28]. In a review article by Batista et al. on multiple studies of exercise and IL-10, the authors concluded that the anti-inflammatory effect induced by AEXT seems primarily to be mediated by IL-10 [29]. In the present study, the effect of AEXT on circulating levels of IL-10 was investigated, to our knowledge, for the first time in an African American population, and the results demonstrated that there was a trend for increased IL-10 subsequent to AEXT, although statistical significance was not achieved. More notably, the results of the present study indicated that in our African American population sample, the significant improvement in cardiovascular fitness, as measured by VO_2max⁡_, was related to the improvement in plasma levels of IL-10. Future research is warranted in order to assess whether further increases in VO_2max⁡_, subsequent to AEXT, elicit a significant improvement in IL-10.

 We previously reported that African American endothelial cells had significantly greater levels of IL-6 protein expression and produced greater amounts of IL-6 in response to TNF-*α*, an inflammatory cytokine [30]. Oxidative stress and inflammation often occur simultaneously [31]. Kalinowski demonstrated that African Americans have increased levels of oxidative stress resulting in endothelial dysfunction when compared to Caucasians [32], and we also previously demonstrated that compared to Caucasian endothelial cells, African American endothelial cells had significantly greater protein expression levels of NADPH oxidase, the principal source of reactive oxygen species in endothelial cells [33]. Together, the work of others and our group suggests a heightened inflammatory and oxidative stress status in African American endothelial cells. Therefore, an intervention that can dampen this condition before endothelial dysfunction develops to the point where it is manifested clinically may be very important. The results from our present study extend upon our previous work that AEXT may be a nonpharmacological treatment modality which may improve endothelial health in middle-to-older-aged African American adults free of overt CVD. 

The positive changes in endothelial and inflammatory biomarkers after AEXT demonstrated in this study may indicate considerable improvement in CVD risk for the African American population. A substantial portion of the CVD risk reduction associated with exercise training cannot be entirely explained by changes in conventional CVD risk factors [34]. It has been suggested that direct effects of exercise on the vessel wall may account for some of the remaining risk factor reduction gap [35].

The participants in the present study had no significant changes in mean blood pressure following AEXT. These findings are in agreement with most studies that measured blood pressure subsequent to AEXT in individuals with relatively normal resting blood pressure levels. In studies on normotensive and/or prehypertensive populations, blood pressure did not significantly change following AEXT in most cases [36, 37]. Conversely, a review by Hagberg et al. reported that blood pressure significantly decreased in 75% of the hypertensive subjects following AEXT [38]. Despite the fact that mean blood pressure did not change in the present study, the endothelial and inflammatory biomarkers measured in this study related to endothelial health and CVD improved considerably. Therefore, the pronounced benefits on CVD risk reduction resulting from AEXT may go beyond simple blood pressure reduction in an African American population as elucidated by the results of the present study. 

Several limitations must be noted when interpreting our study findings. First, our sample size is small, but this was due to the exclusion of diabetics, smokers, participants with CVD, or other chronic diseases and those on medications that affect cardiovascular or renal hemodynamics, on lipid lowering medications, or on more than one antihypertensive medication. This was done to create a more homogenous group and to ensure lack of confounding variables that may influence endothelial or inflammatory marker levels. It should be noted that even with a relatively small sample size, we observed significant changes in three of the four primary outcome measures subsequent to AEXT. Second, because of the observational nature of the study design, we cannot infer mechanisms underlying exercise training induced changes in inflammatory status or endothelial function. Third, there are presently no standardized methods for the measurement of microparticles. Processing and analyzing techniques differ from investigator to investigator, and thus comparisons across studies for EMPs should be done cautiously. Fourth, no control group was included in the study design, and thus it is difficult to ascertain whether the observed changes were exclusively due to AEXT and not to the result of an unidentified confounding factor. Finally, the sample population was predominately female, and thus our findings may have limited generalizability to African American males. 

In conclusion, the results of the present study are novel because to our knowledge, for the first time, FMD%, CD62E+ EMPs, IL-6, and IL-10 have been measured together prior to and subsequent to AEXT in a population of African Americans. The primary findings of the study revealed favorable alterations in the endothelial and inflammatory biomarkers measured subsequent to AEXT. Therefore, aerobic exercise training may be a viable, nonpharmacological method to improve inflammation status and endothelial function and thereby contribute to risk reduction for CVD in African Americans.

## Figures and Tables

**Table 1:** Laboratory values of participants before and after AEXT.

Variable	Participant number	Pre-AEXT	Post-AEXT	Percent change
BMI (kg/m^2^)	*n* = 42	31.4 ± 0.9	30.6 ± 0.9*	−2.5%
VO_2 max_ (mL/kg/min)	*n* = 41	25.9 ± 0.9	28.2 ± 1.1**	8.9%
SBP (mm Hg)	*n* = 41	124.2 ± 1.9	123.6 ± 2.2	−0.5%
DBP (mm Hg)	*n* = 41	78.7 ± 1.1	78.9 ± 1.2	0.3%
Total cholesterol (mg/dL)	*n* = 35	190.9 ± 4.2	190.4 ± 5.2	−0.3%
LDL cholesterol (mg/dL)	*n* = 36	108.7 ± 3.6	111.9 ± 4.3	2.9%
HDL cholesterol (mg/dL)	*n* = 36	66.8 ± 3.3	65.6 ± 3.4	−1.8%
Triglycerides (mg/dL)	*n* = 36	83.0 ± 5.7	70.1 ± 3.3**	−15.5%
Fasting glucose (mg/dL)	*n* = 34	95.1 ± 1.7	88.5 ± 1.8**	−6.9%

Participant number represents usable sample for variables.

Values are expressed as mean ± SEM. BMI: body mass index; SBP: systolic blood pressure; DBP: diastolic blood pressure; HDL: high-density lipoprotein; LDL: low-density lipoprotein.

*Denotes significant differences pre- versus post-AEXT; *P* < 0.05.

**Denotes significant differences pre- versus post-AEXT; *P* < 0.01.
